# Quality of Life After Umbilical Hernia Repair

**DOI:** 10.7759/cureus.19016

**Published:** 2021-10-24

**Authors:** Nadim Malibary, Mahmoud Shurrab, Mohammed O Albariqi, Mohnad Bohairi, Ahmed S Basabain, Mishal Y Alqurashi, Turki A Madani

**Affiliations:** 1 Visceral and General Surgery, King Abdulaziz University Faculty of Medicine, Jeddah, SAU; 2 Medicine, King Abdul-Aziz University Hospital, Jeddah, SAU; 3 Medical Intern, King Abdulaziz University Faculty of Medicine, Jeddah, SAU; 4 Surgery Department, King Abdulaziz University Faculty of Medicine, Jeddah, SAU; 5 Internal Medicine, King Abdulaziz University, Jeddah, SAU; 6 General Surgery, King Abdulaziz University, Jeddah, SAU

**Keywords:** hernia, quilty of life, surgical repair, umbilical hernia, kingdom of saudi arabia (ksa)

## Abstract

Background: Mesh is beneficial in the repair of umbilical hernias. But it may cause chronic pain due to inflammatory reactions, which may impair the patient's quality of life.

Objectives: To assess and compare the quality of life of patients following umbilical hernia repair with and without mesh.

Methods: During the study period, 45 patients underwent umbilical hernia repair. The study was conducted at King Abdul-Aziz University Hospital (KAUH), KSA. Data were collected using medical records, and each patient was contacted by telephone, to fill the “Carolina Comfort Scale (CCS)” survey. The survey assesses the grade of pain, sensation of mesh, and movement limitation in different situations.

Results: A non-significant difference was found between mean quality of life (QOL) scores of mesh and non-mesh groups. The relationship between CCS and gender was not significant in both groups. However, males had significantly higher CCS scores in mesh-treated cases. There was no statistically significant relationship between CCS and comorbidity, nationality, or symptoms. The overall CCS score did not differ statistically between mesh-treated and non-mesh-treated cases.

Conclusion: The CCS score did not differ between mesh-treated and non-mesh-treated cases. It is suggested that future multicentric studies with a larger sample size be conducted.

## Introduction

An umbilical hernia is defined as a midline abdominal wall defect extending from 3 cm above to 3 cm below the umbilicus, and it is a common adult diagnosis, with a global prevalence of 2% [1]. Hernia symptoms include a periumbilical bulge and abdominal pain and discomfort, particularly when coughing, lifting, or bending over. Some people may not be aware of the condition until they have a medical checkup [2].

Pregnancy, weightlifting, constipation, weight gain, chronic cough, damage from an injury or surgery, and abdominal wall defects [3] may cause a hernia. The time it takes for a hernia to develop is determined by its causes, which are muscle weakness and strain. Patients should seek medical attention if there is a painful or noticeable bulge on the abdomen [3]. The patient can feel the bulge by touching the affected area or notice it when standing upright [4]. Ventral hernias are quite common and are said to affect no less than 2% of males, though data from the United States put the figure at 1.5 % (15 cases per 1000) [5].

More than 20 million hernias are operated on around the world yearly [6], and umbilical hernia repairs dominate day-case operating lists [7]. Most symptomatic or clinically visible umbilical hernias require surgical repair, which can be accomplished through suture repair or mesh placement [8].

The World Health Organization (WHO) defines the quality of life (QOL) as an individual’s perception of their position in life in the context of the culture and value systems in which they live, in relation to their goals, expectations, standards, and concerns [9].

The California Comfort Scale (CCS) is a 23-item questionnaire that quantifies the severity of pain, mesh sensation, and movement limitation from the hernia or surgical site during the following eight activities: lying down, bending over, sitting up, activities of daily living, coughing or deep breathing, walking, climbing stairs, and exercise [10,11].

A 1% recurrence rate of large umbilical hernia treated with mesh versus suture repair was found in two retrospective randomized control trials [12]. Umbilical hernias are currently treated with both mesh and suture repair [13].

As the postoperative QOL has become an important outcome measure following herniorrhaphy [9-11], this study aimed to measure the QOL of patients after umbilical hernia repair with mesh and compare them with non-mesh-treated cases.

## Materials and methods

This cross-sectional study was completed from January 2019 to December 2020 at King Abdul-Aziz University Hospital (KAUH), Jeddah, Saudi Arabia. All adult patients who underwent umbilical hernia repair between January 1, 2019, and December 31, 2020, were included in the study.

Ethical approval was obtained from the Unit of Biomedical Ethics Research Committee at King Abdul-Aziz University, Faculty of Medicine (reference no. 443-18). Data were collected using the hospital's electronic medical records, and each patient was contacted by telephone, and after his/her verbal consent, a phone interview was conducted to fill the “Carolina Comfort Scale” survey [11]. The survey is a ready validated survey with different parts: grade of pain, the sensation of mesh, and moving limitation in different situations (i.e., laying down, bending over, sitting up, performing activities of daily living, coughing or deep breathing, walking or standing, walking up or down the stairs, when exercising).

Statistical analysis

After checking the data for errors and completeness, all data were given input to SPSS version 24 (Armonk, NY: IBM Corp.) for analysis. The analysis was performed at a 95% confidence interval. The categorical variables were presented as frequencies and percentages; continuous variables were presented as mean and standard deviation. The mean score for all CCS measuring items was presented. The mean total CCS score was calculated by adding individual CCS items. The mean total CCS was presented and compared between cases with mesh and without mesh, males and females, nationalities, and with comorbidity and without comorbidity groups by Mann-Whitney U test. A non-parametric test was done because the mean total CCS was non-normally distributed. Correlation between age, operation duration, blood loss, and total CCS was done by the Spearman Rank Correlation test. 

## Results

This retrospective chart review study involved a total of 45 cases; 29 (64.4%) were female, and 36 (80%) were Saudi. Of the cases, 40% practiced regular or consistent sports activity. Comorbidity was present in 42.2% of cases. The most common symptom was the feeling of a mass (55.6%). Only four (8.9%) of the cases were smokers. These socio-demographic characteristics for the mesh and non-mesh groups were presented in Table 1.

**Table 1:** Socio-demographic characteristics of mesh group (n = 29)

Characteristics	N (%)/mean ± SD
Gender	
Male	11 (37.9)
Female	18 (62.1)
Nationality	
Saudi	21 (72.4)
Non-Saudi	8 (27.6)
Age in years	48.86 ± 14.82
BMI (kg/m^2^)	31.12 ± 6.41
Activity	
Sedentary	10 (34.5)
Active	18 (62.1)
Very active	1 (3.4)
Comorbidity	
Yes	13 (44.8)
No	16 (55.2)
Symptoms	
Mass	12 (41.4)
Pain	8 (27.6)
Mass and pain both	9 (31.0)
Smoking	
Non-smoker	26 (89.7)
Smoker	3 (10.3)

The mean age and BMI of all cases were 49.33 + 13.15 years and 31.81 ± 7.44 kg/m^2^. The difference between the mesh group and the non-mesh group in terms of age and BMI was not statistically significantly different (p-value 0.618 and 0.799).

Regarding the surgical procedure in cases with mesh (n = 29), 25 (86.2%) had open surgery and elective operation. The commonest mesh used was Parietex composite ventral patch (44.8%), and for 20.7% of patients, the size of the mesh was 10 × 15. Most of the cases did not have any symptoms after one month of the operation (82.8%). In addition, 89.6% did not suffer from any complications (pain, seroma, wound infection) during the post-op follow-up visits. Only 69.0% of the patients suffered from constipation before the surgery, and six patients complained of recurrence 20.7% (Table 2).

**Table 2:** Operation-related variables

Variables	N	%
Type of surgery		
Laparoscopic	4	13.8
Open	25	86.2
Urgency of operation		
Elective	25	86.2
Emergent	4	13.8
Type of the mesh		
Parietex composite ventral patch	13	44.8
Ventralight mesh	4	13.8
Vypro ll mesh	3	10.3
St phasix mesh	3	10.3
Dipromed surgical mesh	2	6.9
Macroporous mesh	2	6.9
Proline mesh	2	6.9
Size of the mesh		
10 × 15	6	20.7
6 × 6	5	17.2
15 × 20	4	13.8
6 × 4	4	13.8
6 × 8	4	13.8
10 × 12	3	10.3
15 × 15	1	3.4
6 × 11	1	3.4
6 × 12	1	3.4
Fixing method		
Thread	20	69.0
Tuckers	7	24.1
Both	2	6.9
Symptoms after one month		
None	24	82.8
Pain	2	6.9
Pain + discomfort	2	6.9
Pain + mass	1	3.4
Have constipation before surgery		
No	20	69.0
Yes	9	31.0
Follow-up complications		
None	26	89.6
Pain	1	3.4
Seroma	1	3.4
Wound infection	1	3.4
Recurrence hernia		
No	23	79.3
Yes	6	20.7

The mean difference between scores of CCS questionnaire items of cases with mesh and without mesh was not statistically significant except for item numbers 10, and 22 (p-values=0.010 and 0.023), respectively. 

The relationship between CCS and gender was not significant for all cases with and without mesh. But for the mesh group, males had significantly higher CCS of 10.91 ± 14.52 as opposed to females 7.56 ± 11.21 (p-value=0.011; Table 3). The relationship between CCS and the presence of comorbidity was not statistically significant (Table 4). Similar results were seen between the CCS and nationality relationship, and CCS and symptoms relationship t (all p-values>0.050). 

**Table 3:** Mean and standard deviation of all individual CCS questions (mesh group) CCS: Carolina Comfort Scale

Q. No.	Questions	Mean	Std. Deviation
1	While laying down, do you have a sensation of mesh?	0.2069	0.77364
2	While laying down, do you have pain?	0.7586	1.50369
3	While bending over, do you have a sensation of mesh?	0.5357	1.10494
4	While bending over, do you have pain?	0.7586	1.27210
5	While bending over, do you have movement limitations?	0.6897	1.53770
6	While sitting up, do you have a sensation of mesh?	0.0345	0.18570
7	While sitting up, do you have pain?	0.3103	0.84951
8	While sitting up, do you have movement limitations?	0.2414	0.73946
9	"While performing activities of daily living (getting out of bed, bathing, getting dressed), do you have a sensation of mesh"?	0.0690	0.25788
10	"While performing activities of daily living (getting out of bed, bathing, getting dressed), do you have pain"?	0.2069	0.55929
11	While performing activities of daily living (getting out of bed, bathing, getting dressed), do you have movement limitations?	0.2069	0.49130
12	When coughing or deep breathing, do you have a sensation of mesh?	0.3448	0.76885
13	When coughing or deep breathing, do you have pain?	0.9310	1.22273
14	When coughing or deep breathing, do you have movement limitations?	0.6207	1.23675
15	When walking or standing, do you have a sensation of mesh?	0.3448	0.76885
16	When walking or standing, do you have pain?	0.3448	0.76885
17	When walking or standing, do you have movement limitations?	0.3793	1.11528
18	When walking up or downstairs, do you have a sensation of mesh?	0.5185	1.34079
19	When walking up or downstairs, do you have pain?	0.6429	1.44566
20	When walking up or downstairs, do you have movement limitations?	0.5000	1.31937
21	When exercising (other than work-related), do you have a sensation of mesh?	0.2083	1.02062
22	When exercising (other than work-related), do you have pain?	0.0417	0.20412
23	When exercising (other than work-related), do you have movement limitations?	0.0833	0.40825

**Table 4:** Relationship between CCS (mesh group) and gender; CCS and comorbidity CCS: California Comfort Scale

Variables	Cases	N	CCS mean	CCS standard deviation	p-value
Gender	Male	11	7.25	13.70	0.620
	Female	18	3.13	3.09	
Comorbidity	Yes	13	3.29	3.99	0.837
	No	16	5.13	9.74	

Correlation between age, BMI, blood loss, operation duration, and CCS score were checked for the mesh group. Moderate strength positive correlation was found between blood loss and BMI (r=0.619, p-value≤0.001). Weak positive correlations were found between age and BMI (p=0.001), age and operation duration (p=0.022), and BMI and operation duration (p=0.002; Table 5). No statistically significant relationship was found between the mesh group and no mesh group CCS (p=0.905; Table 6).

**Table 5:** Correlation between age, BMI, blood loss during the operation, and CSS (cases with mesh) CCS: California Comfort Scale; BMI: body mass index *Means correlation is significant at the 0.01 level (two-tailed)

		Age	Operation duration	Blood loss	Total CCS
Age	Correlation coefficient	1.000	0.423^*^	0.370^*^	−0.493^*^
	p-value	.	.022	.048	.017
Operation duration	Correlation coefficient		1.000	.365	.005
	p-value		.	.051	.984
Blood loss	Correlation coefficient			1.000	−.182
	p-value			.	.407

**Table 6:** Total CCS of all cases mesh and no mesh groups CCS: California Comfort Scale

Cases	N	CCS mean	CCS standard deviation	p-value
With mesh	29	4.57	8.35	0.720
Without mesh	14	3.00	1.41	

## Discussion

The purpose of this study was to compare the QOL of patients after umbilical hernia repair with and without mesh. The current study discovered that the most common symptom was a sense of mass (55.6%). This rate is lower than that found in a previous study of 300 patients from 12 Italian hospitals (76.3%) [13].

In the mesh group, 86.2% of patients underwent open surgery and elective surgery. A previous study used the CCS to assess changes in health-related QoL overtime after different mesh hernia repair procedures yielded different results. In the former study, all umbilical hernia repairs were performed laparoscopically [14]. In another study done in Denmark, from the 990 mesh group studied, 724 patients (73.1%) had open surgery [15].

In the present work, we mostly used absorbable mesh (44.8%). A randomized study was done to compare the effect of non-absorbable tackers (NAT) versus absorbable tackers for laparoscopic incisional and ventral hernia repair (LIVHR). The study discovered that NAT is a cost-effective method of mesh fixation in LIVHR patients, with comparable early and late postoperative outcomes in terms of pain, QOL, and patient satisfaction scores [16].

In this study, we discovered that the majority of cases (79.1%) were repaired electively, while the remaining (20.9%) were repaired as emergency cases. In a previous Saudi study conducted at King Fahd Hospital (KFH), open surgery was used in 89.7% of cases and laparoscopy was used in 10.3%, while mesh was used in 46.3% of all cases [17]. In the previously mentioned Denmark study, open repair (92.2%) and only 7.8% had laparoscopic mesh [15].

After one month of the operation, two patients (6.9%) complained of pain after one month of surgery, two patients (6.9%) complained of pain associated with discomfort, and one patient (3.4%) complained of pain associated with mass. Comparison between the occurrence of postoperative pain between mesh and suture repair during all-time points was done in a previous study. It was revealed that two weeks after the operation, 102 patients (74%) in the suture repair group and 111 patients (76%) in the mesh group were free of pain with no significant difference. After two years, 129 patients (93%) in the suture repair group and 138 patients (95%) in the mesh group were free of pain [18]. Another study found that pain was reported by 70% of patients in the suture repair group and in 68% in the mesh group [13].

In terms of QOL among this study's patients, the mean difference between patients with and without mesh was not statistically significant. A different result was revealed in a Danish study, where the CCS scores changed significantly, indicating that health-related QoL gradually improved after four different types of mesh hernia repair over the first three months [14]. Another study, on the other hand, compared QoL at baseline preoperatively and 12 months after umbilical repair and discovered a non-significant difference [18]. Kaufmann et al. discovered that mesh repair for small umbilical hernias (diameter 1-4 cm) reduced the number of recurrences significantly more than suture repair [13,18].

Although the overall complication rate in the laparoscopic group is twice that of the open group, and the length of hospital stays and readmission tended to be higher after a laparoscopic repair, our data do not suggest a significant difference in outcome following laparoscopic rather than open repair, umbilical rather than epigastric hernia repair, mesh or no mesh repair. This agrees with the study done in Denmark, where no important difference was found in the outcome results regarding open umbilical and epigastric hernia repair, and mesh or no mesh [15]. However, our study included a much smaller sample.

In 2018, a study demonstrated that laparoscopic umbilical hernia repair results in less operative time, faster postoperative recovery, and a higher QOL than open umbilical hernia repair [18]. Another systematic review and meta-analysis observed that mesh repair of umbilical hernia protected significantly against recurrence compared with a non-mesh sutured repair, and the use of mesh repair did not increase the risk of surgical site infection, seroma formation, hematomas, or chronic pain [19].

The study's findings revealed that the relationship between CCS and gender was not significant for mesh and non-mesh cases. Males, on the other hand, had significantly higher CCS score in the mesh group. This coincides with our results, as looking at CCS, predictors of outcome, and recurrence and reoperation rates during the first postoperative year showed that an event-free recovery was associated with male sex and laparoscopic surgery [20].

The relationship between CCS and the presence of comorbidity was not statistically significant in all cases in the current study, as well as in the mesh group versus the no mesh group. In terms of the recurrence component of QOL, a previous study discovered that BMI had no effect on the number of recurrences [13].

Umbilical hernias have a higher morbidity and mortality rate than inguinal hernias due to the increased risk of incarceration and strangulation, which necessitate emergency repair [21]. In the present study, most cases (82.8%) did not have symptoms one month after the operation. Besides, 89.6% did not have complications during the follow-up visits. A previous study found that the frequency and severity of symptoms were significantly higher in the laparoscopic ventral hernia repair group than in the open ventral hernia repair group [19]. Another study found that the most common reason for crossover to mesh repair in the non-mesh group was comorbidity of the patient (high body-mass index [BMI] and heavy occupational lifting) [13]. The positive correlations found between BMI and operation duration in the present work were also observed in previous studies [20,22].

Limitations

The cross-sectional design of the current study, which could reveal the association between variables but not the causal association, is an important limitation. As a result, future prospective studies are advised. Other limitations were the small sample size, the short follow-up period, being a single-center study, and the possibility of a recall bias.

## Conclusions

Our study found a non-statistical significant relationship between CCS scores and the presence of comorbidity or presenting symptoms. The overall CCS score did not differ statistically between the mesh and non-mesh groups, however, males of the mesh group had a better CCS score compared to the no mesh group. Multicentric studies with a larger sample size are recommended.
